# From repair to risk: ICC-like fibrosis following 3D bioprinted bile duct reconstruction – a letter to the editor

**DOI:** 10.1097/MS9.0000000000004436

**Published:** 2025-11-26

**Authors:** Mashal Naveed, Minahil Amjad, Menahil Fazal, Mawra Naveed, Mahnoor Jan, Kamil Ahmad Kamil

**Affiliations:** a Allama Iqbal Medical College, Lahore, Pakistan; b Shaikh Khalifa Bin Zayed Al-Nayhan Medical and Dental College, Lahore,Pakistan; c Shaikh Khalifa Bin Zayed Al Nahyan Medical and Dental College, Lahore, Pakistan; d Internal Medicine Department, Mirwais Regional Hospital, Kandahar, Afghanistan

**Keywords:** 3D bioprinting, bile duct regeneration, biodegradable polymeric stents, cholangiocarcinoma-like fibrosis, fibroinflammatory response

## Abstract

Three-dimensional bioprinting integrates computing technology to precisely construct living tissues from cells, biomaterials, and growth factors. It is being used specifically in bile duct regeneration through the application of biodegradable polymers. However, recent studies indicate that the degradation of these polymers may result in inflammation and fibrosis similar to that observed in intrahepatic cholangiocarcinoma. These findings underscore the need for standardized bioink formulations, careful monitoring and integrated histological and molecular follow up to prevent diagnostic confusion between regenerative fibrosis and malignancy.

*To the Editor*,

Three-dimensional (3D) bioprinting is a computer-aided process that assembles cells, growth factors, bioinks, and biomaterials layer by layer to produce complex 3D biological constructs. Bile duct tissue regeneration has advanced through scaffolds of biocompatible materials like polycaprolactone and poly (lactic *co*-glycolic acid), frequently combined with living cells to aid regrowth^[1]^. However, emerging studies suggest that such bioprinted polymeric stents can induce inflammation and fibrosis, causing complications that resemble the malignant transformation of intrahepatic cholangiocarcinoma (ICC) (a primary liver cancer that arises from bile duct epithelial cells)^[2]^.

Recent clinical investigations indicate that polymer degradation byproducts can acidify the biliary milieu, inducing localized ductal inflammation^[1]^. The resulting chronic fibroinflammatory signaling, primarily driven by interleukin-6 (IL-6) and tumor growth factor beta-1 (TGF-β1) pathways, results in aberrant ductal growth and stromal desmoplasia, characteristics of ICC-like lesions^[3]^. The lesions are histologically identified by heavy collagen deposition and myofibroblastic activation mimicking early neoplastic alteration, despite lacking true malignancy. Such ICC-like fibrosis has been reported in long-term implant follow-ups during preclinical bile duct reconstruction experiments^[2]^. These findings highlight the importance of a proper clinical investigation of 3D bioprinted models to prevent false diagnoses.

Conventionally, non-biodegradable stents were used, which, although effective initially, led to long-term complications such as stent restenosis. Consequently, repeated interventions for stent removal or replacement were needed. In contrast, biodegradable stents, as shown by Lee *et al* in their study, are engineered to provide temporary support and they safely degrade, eliminating the need for further interventions^[4]^. However, Yan *et al* highlighted in their study that the degradation of biodegradable stents can provoke an inflammatory and fibrotic response that mimics the desmoplastic microenvironment of ICC^[5]^. The chronic inflammation and epithelial hyperplasia observed with biodegradable stents can be misinterpreted as ductal atypia and aggressive stromal remodeling, which, if not distinguished from true neoplasia, risk misdirected oncological therapy^[2]^. This suggests that regenerative therapy may paradoxically induce ICC-like fibrosis, representing a critical limitation of advanced biodegradable stent technology.

Despite the growing interest in bioprinted biliary scaffolds, the available evidence regarding them remains limited to preclinical studies, with long-term human trials lacking. Consequently, the differentiation between observed fibrosis changes and ICC remains ambiguous. Divergences in bioink composition, scaffold rigidity, and cellular makeup complicate the assessment and consistency of fibrosis outcomes^[6]^. Additionally, the histological similarity between dysregulated fibrosis remodeling and ICC poses significant diagnostic difficulties^[7]^. Ethical and logistical barriers limit the follow-up crucial for evaluating the fibrosis, which further invalidates the diagnosis.

Despite 3D bioprinting’s transformative potential for bile duct reconstruction through noninvasive tissue regeneration, a paradoxical result was seen in preclinical tests: the degradation of the polymer can turn the area where it was implanted acidic, which in turn activates IL-6 and TGF-β1 that cause a prolonged inflammation that is followed by deposition of dense collagen, activation of myofibroblasts and stromal desmoplasia; all these changes are histologically similar to early ICC and still the malignancy is not present^[1–3]^. This ICC-like fibrosis, which has been reported in long-term implant studies but not in human trials, poses a danger of mixing up the processes of regenerative remodeling and neoplastic progression, and hence is likely to result in false oncological interventions^[2,5]^. In order to avoid such diagnostic errors and facilitate the safe translation of the findings, the researchers will have to prescribe the bioink formulations to be used with strict tests for scaffold rigidity^[6]^ integrate surgical-histological-molecular monitoring in extended preclinical cohorts^[7]^ and speed up the ethically designed longitudinal human studies to clearly distinguish between benign fibrotic adaptation and pathologic transformation^[2]^.

In summary, 3D bioprinting demonstrates considerable potential for biliary tract reconstruction, yet its ability to trigger ICC-like fibrosis underscores the need for standardized testing and extended monitoring. Future studies should merge surgical, histological, and molecular follow-up data for safer clinical translation of 3D molecular bioprinting.

This letter to the editor adheres to the TITAN Guidelines 2025 on transparency, integrity, and the use of artificial intelligence in medical writing and publishing^[8]^.

## Data Availability

Not applicable.
